# The B-Cell Follicle in HIV Infection: Barrier to a Cure

**DOI:** 10.3389/fimmu.2018.00020

**Published:** 2018-01-25

**Authors:** Matthew P. Bronnimann, Pamela J. Skinner, Elizabeth Connick

**Affiliations:** ^1^Division of Infectious Disease, Department of Medicine, University of Arizona, Tucson, AZ, United States; ^2^Department of Veterinary and Biomedical Sciences, University of Minnesota, St. Paul, MN, United States

**Keywords:** B cell follicle sanctuary, follicular dendritic cell, gamma delta T cells, NK cells, cytotoxic T-cell (CTL), HIV cure research, T follicular helper cell subsets, regulatory T cells

## Abstract

The majority of HIV replication occurs in secondary lymphoid organs (SLOs) such as the spleen, lymph nodes, and gut-associated lymphoid tissue. Within SLOs, HIV RNA^+^ cells are concentrated in the B-cell follicle during chronic untreated infection, and emerging data suggest that they are a major source of replication in treated disease as well. The concentration of HIV RNA^+^ cells in the B-cell follicle is mediated by several factors. Follicular CD4^+^ T-cell subsets including T-follicular helper cells and T-follicular regulatory cells are significantly more permissive to HIV than extrafollicular subsets. The B cell follicle also contains a large reservoir of extracellular HIV virions, which accumulate on the surface of follicular dendritic cells (FDCs) in germinal centers. FDC-bound HIV virions remain infectious even in the presence of neutralizing antibodies and can persist for months or even years. Moreover, the B-cell follicle is semi-immune privileged from CTL control. Frequencies of HIV- and SIV-specific CTL are lower in B-cell follicles compared to extrafollicular regions as the majority of CTL do not express the follicular homing receptor CXCR5. Additionally, CTL in the B-cell follicle may be less functional than extrafollicular CTL as many exhibit the recently described CD8 T follicular regulatory phenotype. Other factors may also contribute to the follicular concentration of HIV RNA^+^ cells. Notably, the contribution of NK cells and γδ T cells to control and/or persistence of HIV RNA^+^ cells in secondary lymphoid tissue remains poorly characterized. As HIV research moves increasingly toward the development of cure strategies, a greater understanding of the barriers to control of HIV infection in B-cell follicles is critical. Although no strategy has as of yet proven to be effective, a range of novel therapies to address these barriers are currently being investigated including genetically engineered CTL or chimeric antigen receptor T cells that express the follicular homing molecule CXCR5, treatment with IL-15 or an IL-15 superagonist, use of bispecific antibodies to harness the killing power of the follicular CD8^+^ T cell population, and disruption of the follicle through treatments such as rituximab.

## The B-Cell Follicle in Untreated and Treated Disease

HIV and SIV RNA^+^ cells are concentrated in B-cell follicles of secondary lymphoid organs (SLOs) during chronic untreated infection prior to the development of AIDS (1–3). In chronic HIV disease, a follicular CD4^+^ cell is ~30–40-fold more likely to harbor HIV RNA than an extrafollicular CD4^+^ cell (2, 4). The follicular concentration of RNA^+^ cells has only been examined in lymph nodes (LNs) during HIV infection, but it has also been observed in other SLOs such as the spleen, and gut-associated lymphoid tissue (GALT) during chronic SIV infection of rhesus macaques (3). Notably, in advanced disease, the follicular concentration of virus-producing cells wanes, and lymphoid tissue architecture is increasingly disrupted demonstrating follicular depletion and involution (3, 5). During antiretroviral therapy (ART), the frequency of HIV RNA^+^ cells in LNs and B-cell follicles is reduced and lymphoid architecture is at least partially restored (6). However, RNA^+^ cells are still detected in B-cell follicles during ART in both HIV and SIV infection (7–9). It remains controversial whether HIV RNA^+^ cells found in SLOs during ART are the result of successful new infection or reactivation of latently infected cells. It has been reported that ART concentrations are suboptimal in LNs, which may allow continued cycles of replication (10). However, evidence of ART resistance mutations arising from SLOs of well-suppressed patients is lacking, as might be expected with suboptimal ART concentration (6, 11, 12). Regardless, after ART cessation, viral rebound occurs in SLOs in both HIV and SIV infection (13, 14). Given the concentration of HIV/SIV RNA^+^ cells in the B-cell follicle during ART, it seems likely that the HIV reservoir in B-cell follicles contributes to viral rebound post-ART cessation.

## Susceptibility of Follicular Helper T-Cells to Infection

The dominant follicular CD4^+^ T-cell subset in B-cell follicles is T-follicular helper cells (TFH). TFH are essential for the development of germinal center (GC) reactions and the subsequent affinity maturation, class switching, and differentiation into memory subsets of B-cells (15). TFH are defined by expression of the master transcription factor BCL-6, constitutive expression of the follicular homing receptor CXCR5, and production of IL-21, which mediates B-cell class switching (16–21). Interestingly, during HIV infection, TFH cells expand numerically, but have diminished function (22, 23). TFH also serve as a major reservoir of HIV and SIV RNA^+^ and DNA^+^ cells during chronic infection (2, 4, 22, 24). TFH are more susceptible to HIV infection than extrafollicular (EF) CD4^+^ T-cell subsets *ex vivo* (25–27). It should be noted that the relative susceptibility of TFH to HIV infection has mainly been determined by spinoculation. While spinoculation is an efficient way to facilitate HIV/lentiviral infection, it is not necessarily representative of *in vivo* infection pathways. During chronic HIV infection, TFH reside in close proximity to follicular dendritic cells (FDCs) decorated with HIV-immune complexes (ICs), which may serve as a major route of infection *in vivo*. Importantly, TFH are also more susceptible to FDC-bound HIV-IC-mediated infection than EF subsets (28).

T-follicular helper cells can be further subdivided into a GC-localized subset defined by high expression of PD-1, and a non-GC subset defined by intermediate or low expression of PD-1. GC TFH are even more susceptible to HIV infection than non-GC TFH *ex vivo* (25). This finding was supported with the observation that *in vivo* HIV RNA^+^ cells are more concentrated in GCs than non-GC areas of the B-cell follicle, or EF regions (25). More recently, a T-follicular regulatory subset of CD4^+^ T cells (TFR) was discovered in humans (29–31). TFR limit the GC reaction and antibody production to prevent autoimmunity (31). Chronic HIV and SIV infection cause TFR to expand numerically (32). *Ex vivo*, HIV infection causes TFR to increase expression of regulatory molecules and more potently inhibit TFH function (32). It is therefore likely that HIV-related TFR expansion contributes to poor TFH function in HIV-infected individuals. TFR are also more susceptible to R5-tropic HIV infection *ex vivo* and contain the highest percentage of HIV RNA^+^ cells compared to EF, TFH, and EF Treg CD4^+^ subsets (26).

Several mechanisms have been proposed to be related to increased permissivity of TFH and TFR. TFH have been shown to have increased expression of the antiapoptotic protein BCL-2 when infected with R5-tropic HIV compared with EF CD4^+^ T-cell subsets (33). Furthermore, the TFH master transcription factor BCL-6 mediates constitutively diminished expression levels of interferon-stimulated genes important in antiviral immunity (27). The enhanced susceptibility of TFR compared to TFH to HIV R5-tropic infection is likely partially mediated by higher levels of CCR5 expression and an enhanced proliferative state (26).

### TFH Memory

It is well established in peripheral blood that CD4 central memory (CM) and transitional memory subsets contain the majority of proviral HIV DNA during ART (34). During chronic untreated HIV and SIV infection memory, TFH in LNs are enriched for DNA^+^ cells (22, 35). Recently, it was reported in HIV infection that during ART, PD-1^+^ memory TFH are the major reservoir of cells harboring replication competent virus (36). Similarly, during SIV infection, ART results in the concentration of SIV DNA^+^ cells in PD1^+^ CTLA-4^+^ TFH in the B cell follicle. Interestingly, in the T cell zone, ART resulted in the concentration of SIV DNA in PD-1^−^ CTLA4^+^ cells (37). The extremely low number of memory SIV DNA^+^ cells that could be isolated prevented detailed quantification of the relative contribution of PD-1^+^ CTLA-4^+^ and PD-1^−^ CTLA-4^+^ memory CD4 T cells to the pool of replication competent SIV. However, replication competent virus was detected in PD-1^+^ CTLA-4^+^ memory CD4 T cells in seven of seven animals and in PD-1^−^ CTLA-4^+^ memory CD4^+^ T cells in six of seven animals (37). Taken together, these data suggest that memory PD-1^+^ TFH contain a large reservoir of replication competent HIV and SIV during ART.

Interestingly, a recent study demonstrated that peripheral blood TFH (pTFH) constitute the major reservoir for replication competent HIV from peripheral blood CM CD4^+^ T cells of patients on ART (38). Furthermore, pTFH were more susceptible than non-pTFH peripheral blood CD4 T cell subsets to HIV infection *ex vivo* (38). The existence of pTFH seems like an oxymoron, given the close association of TFH with the B-cell follicle. However, pTFH express CXCR5 and are capable of stimulating B cell antibody production *via* IL-21, in a manner analogous to TFH (39). How TFH and pTFH are related in origin and function in healthy or HIV-infected individuals remains largely unknown. In mice, some pTFH appear to traffic to GCs and appear similar to TFH post immunization, suggesting that cells may be able to transition between TFH and pTFH (40).

### Impairments in Humoral Immunity Mediated by TFH Dysfunction

The majority of HIV-infected individuals fail to produce broadly neutralizing antibodies (bnAbs) (41). Most bnAbs show evidence of somatic hypermutation (42). Given the critical importance of TFH to the GC reaction and resulting somatic hypermutation, it seems likely that HIV infection in the B cell follicle and the resulting changes in frequency and function of TFH and TFR contributes to the inability of most individuals to produce bnAbs. This hypothesis is supported by the observation that the induction of bnAbs in HIV-infected individuals correlated with the frequency of pTFH (43). Additionally, in rhesus macaques vaccinated with HIV envelope trimers, the quality of TFH in GCs was associated with the production of bnAbs (44). Furthermore, in SHIV-infected rhesus macaques, TFH frequencies were associated with more IgG^+^ B cells in GCs and bnAb production (45). Finally, it is well established that HIV-infected individuals have poor antibody responses to vaccines (46). It was recently demonstrated in patients on ART that pTFH function correlated with the ability to respond to the H1N1/09 influenza vaccine (47). Taken together, these data suggest that defects in TFH, mediated by direct infection and/or TFR expansion, may cause systemic defects in humoral immunity.

## CTL Control

There is extensive evidence that effective cytotoxic T-cell (CTL) responses are critical to controlling HIV and SIV infection. The decline in viremia that occurs following acute infection correlates with the rise of HIV-specific CTL (48). Additionally, several MHC class I alleles are strongly correlated with long-term non-progression and CTLs from long-term non-progressors show enhanced antiviral activity compared to viremic individuals (49, 50). Finally, CD8^+^ T cell depletion during SIV infection results in a spike in viremia (51, 52). Thus, CTL are essential for control of HIV replication, although in most instances unable to fully suppress it.

Early in the epidemic, it was recognized that increased numbers of CD8^+^ cells, many of which are virus-specific CTL, are found within secondary lymphoid tissues including B cell follicles in HIV/SIV-infected individuals compared to uninfected individuals (53–57). Indeed, infiltration of CD8^+^ T cells into B cell follicles was suggested to be a hallmark of HIV infection (58). Nevertheless, frequencies of virus-specific CTL within B cell follicles have been shown to be consistently lower than outside of the follicles (3, 4, 59–61). During acute infection before the virus-specific CTL response has matured, frequencies of HIV RNA^+^ cells per mm^2^ tissue are similar in extrafollicular and follicular areas. Only during chronic infection, after the maturation of the CTL responses does the concentration of HIV RNA^+^ cells in the B cell follicle become apparent (3). CD8 depletion of chronically infected SIV-infected rhesus macaques demonstrated that most of the increase in SIV RNA^+^ cells occurred in the extrafollicular zones, with relatively smaller increases within the follicles (62). Collectively, these findings suggest that CTLs are highly effective in suppressing HIV/SIV replication in EF areas, but not follicular areas and that the latter are relatively immune privileged sites.

While it is clear that there are significantly fewer CD8^+^ T cells in follicular areas, it is unclear whether the CD8^+^ T cells that do localize to B cell follicles have effective cytolytic potential. There is some evidence that CD8^+^ T cells that localize to B cell follicles are on average less cytolytic than others. In SIV infection, follicular CD8^+^ T cells were often in contact with Tregs, and many others expressed PD-1, suggesting they may be functionally exhausted (62). In disaggregated human tonsil cells, ~90% of CD8^+^ T cells with a follicular phenotype (CXCR5^+^CCR7^−^) appear to be part of the newly described CD8^+^ follicular Treg subset, This subset is defined phenotypically as CD44^hi^CXCR5^hi^, and functionally by expression of IL-10, TGF-β, and Tim-3 and low levels of perforin (63) Follicular CD8^+^ Tregs have been demonstrated in mouse models to inhibit TFH expansion, antibody production, and autoimmunity (64, 65). CD8^+^ follicular Tregs suppress TFH-mediated IL-21 and antibody production in response to *ex vivo* HIV infection (63). However, it is important to note that CD8^+^ follicular Tregs have some ability to inhibit HIV replication in TFH *ex vivo*, through unknown mechanisms (63). Additionally, other reports have found that follicular CD8 T cells have potent cytolytic potential against HIV RNA^+^ cells. It was recently reported by He et al. that in peripheral blood from HIV-infected individuals CXCR5^+^ CD8^+^ T cells expressed lower levels of exhaustion markers including Tim-3 and higher levels of IFN-γ than CXCR5^−^ CD8^+^ T cells (66). Importantly, the frequency of CXCR5^+^ CD8^+^ T cells inversely correlated with viral load. Additionally, in the LNs of HIV-infected individuals CXCR5^+^ CD8^+^ T cells expressed higher levels of perforin than CXCR5^−^ CD8^+^ T cells (66). This may suggest that although CXCR5^+^ CD8^+^ T cells are a relatively rare population, they may be more potently cytolytic than CXCR5^−^ CD8^+^ T cells. Interestingly, Petrovas et al. also found that follicular CD8^+^ T cells from LNs of HIV-infected individuals had higher cytolytic activity that extrafollicular CD8^+^ T cells (67). However, they also reported that follicular CD8^+^ T cells had lower polyfunctional cytokine expression and higher expression of PD-1 than extrafollicular CD8^+^ T cells (67).

In summary, the role of follicular CXCR5^+^ CD8^+^ T cells in HIV infection is still unknown. Some studies have suggested that follicular CXCR5^+^ CD8^+^ T cells are potently cytolytic, while others have suggested that the majority of follicular CXCR5^+^ CD8^+^ T cells are part of a CD8^+^ Treg subset that exhibit poor cytolytic control of HIV producing cells and may contribute to defects in humoral immunity. The ability of follicular CD8^+^ T cells to control HIV infection is an area requiring further elucidation.

The mechanisms mediating poor CTL accumulation and possibly poor cytolytic activity in B cell follicles remain poorly understood. However, it is known that for B cells and TFH to localize to the B cell follicle, they must express the follicular homing molecule CXCR5, which is attracted by its ligand CXCL13, which is expressed in B cell follicles. Furthermore, to migrate to the follicle TFH must downregulate CCR7, whose ligands CCL19 and CCL21 lie in the extrafollicular regions (68). Very few HIV- and SIV-specific CTL exhibit the follicular homing phenotype CXCR5^+^CCR7^−^ during chronic infection (3) (and unpublished observations). The mechanisms mediating CXCR5^+^CCR7^−^ CTL development are still poorly understood. However, this phenotype was recently demonstrated in mice to be partially dependent on BCL-6, which is traditionally thought of as the CD4^+^ TFH master transcription factor (61).

## Follicular Dendritic Cells

Follicular dendritic cells accumulate ICs bound to Fc and complement receptors on their cell surface. FDC-bound ICs act as long-term storage for antigen, which is critical to the development of high affinity antibodies including neutralizing antibodies (69). During chronic HIV infection, antibody and/or complement bound HIV-ICs accumulate on FDCs. HIV-ICs bound to the surface of FDCs are known to be present in LNs, GALT, and spleens and are a reservoir of HIV virions of considerable magnitude (70–72). The total amount of HIV RNA stored on FDCs has been estimated to be ~10–40 times greater than the amount of HIV RNA in infected lymphocytes in untreated disease at steady state (73). Additionally, the FDC HIV reservoir persists even during ART, although at diminished levels (9, 73, 74). The necessary time for infectious HIV-IC to be cleared from FDCs during ART is a question critically important to researching a cure. To our knowledge, no reports have demonstrated FDC networks completely free of HIV virions. Years or decades of ART may be necessary to clear HIV-ICs. Alternatively, the presence of low numbers of RNA^+^ cells in SLOs during ART may continually reseed FDCs with HIV-ICs at a low rate and prevent FDC networks from ever being completely cleared of HIV-ICs during ART.

HIV virions bound to FDCs have been shown *ex vivo* to be potently infectious (28, 75, 76). FDC-bound HIV virions are difficult to neutralize and can remain viable *ex vivo* for at least 25 days (75, 76). In an elegant study from Dr. Burton’s laboratory, mice were passively immunized with a non-neutralizing anti-gp41 antibody to allow the formation of ICs upon viral challenge. Upon challenge with HIV, HIV-ICs decorated FDCs in the LNs of these mice (75). Since murine CD4^+^ T cells are non-permissive for HIV infection, the FDCs could not be continually reseeded with HIV-ICs. The FDCs were then isolated at different times post challenge and co-cultured with susceptible human CD4^+^ T cells. The murine FDCs were able to retain infectious virions for at least 9 months (75). This duration of infectivity is remarkable considering that the infectivity of HIV virions is limited by gp120 shedding, mediated by the non-covalent nature of the gp120 gp41 complex and/or by interaction with soluble CD4 (77, 78). The shedding of gp120 by HIV virions is reduced when the virions are in ICs and bound to FDCs, but not individually (79). This suggests that the combination of IC formation and binding to FDCs preserves and protects HIV virions and prevents gp120 shedding. However, the mechanisms of this protection remain completely uncharacterized. One recent report *in vitro* suggests that FDCs endocytose HIV-IC into non-degradative compartments and that this may contribute to protection of the virions (80). It should be noted that several studies using electron microscopy from HIV-infected lymphoid tissue have reported qualitatively that HIV virions reside mostly on the surface of the FDCs (56, 72, 81, 82). However, to our knowledge, the amount of HIV-ICs on the surface of FDCs versus the amount of HIV-ICs in endosomes within FDCs has not been quantified. Additionally, even if the majority of HIV-IC are on the FDC surface at steady state *in vivo*, it is possible that HIV virions are occasionally endocytosed and recycled to the cell surface.

Follicular dendritic cells may also contribute to HIV pathogenesis by release of cytokines. TNF-α produced by FDCs results in a significant increase in HIV replication when co-cultured with infected CD4^+^ T cells (28). Additionally, exposure of FDCs to HIV *in vitro* results in the production of cytokines that are unfavorable to B cell survival and antibody production (83).

## γδ T Cells

γδ T cells are thymus derived cells that create TCRs by VDJ recombination, although with a much reduced diversity compared to αβ T cells (84). γδ T cells have been implicated in tumor immunity as well as microbial immunity (85). The precise ligands of γδ TCRs and the manner in which these ligands are presented is an area of intensive study. However, evidence is mounting that non-peptide pyrophosphate molecules, commonly called phosphoantigens, are a ligand of some γδ TCRs, although likely not the only class of ligand (86, 87). Phosphoantigens are produced in high concentration by both prokaryotes and transformed eukaryotic cells (86).

In adult humans, between 0.5 and 16% of CD3^+^ cells in peripheral blood are γδ T cells (84). In humans there are two major subsets of γδ T cell, Vδ1 and Vδ2. In uninfected humans, the Vδ2 subset is predominant, but during chronic HIV infection, the Vδ2 subset is depleted and the Vδ1 subset is expanded, becoming the predominant subset (88). Recently, it was reported that Vδ2 γδ T cells contain a reservoir of replication competent virus in patients on ART (89). In 14 of 18 patients tested, replication competent virus was recoverable from Vδ2 γδ T cells from peripheral blood (89). Freshly isolated γδ T cells from healthy adults have no or very low CD4 expression, which seemingly should preclude them from HIV infection. However, it has been demonstrated *in vitro* that immune activation can induce CD4 expression and thus susceptibility to infection. In support that this may occur *in vivo*, γδ T cells from patients acutely infected with HIV express considerably more CD4 (range, 9.5–15.9% CD4^+^) compared to uninfected patients (<0.3%) (89).

The ability of γδ T cells to contribute to HIV control is unclear. Several studies have demonstrated lytic properties against HIV-infected cells *in vitro* (90, 91). However, the *in vivo* significance of γδ T cells to control HIV remains less clear. One study reported that in SIV infection a higher percentage of γδ T cells in blood and the endocervix correlated with lower viremia (92).

Almost nothing is known about the ability of γδ T cells to be productively infected or to control HIV infection in SLOs during treated or untreated HIV/SIV infection. However, it is worth noting that γδ T cells have been reported to be able to adopt a TFH-like phenotype *in vitro*. These γδ T cells express BCL-6 and CXCR5 and are capable of stimulating antibody production in B cells (93, 94). In human, tonsils ~50% of Vδ2 γδ T cells express CXCR5 and appear to be able to stimulate antibody production, suggesting that the TFH-like phenotype of γδ T cells may exist *in vivo* (95). It remains unknown if these TFH-like γδ T cells play any role in HIV control or persistence. However, it is tempting to speculate that TFH-like γδ T cells may have increased susceptibility to HIV infection similar to TFH αβ T cells. The role of γδ T cells in HIV persistence and control in SLOs and B cell follicles in particular is an area ripe for further study.

## NK Cells

NK cells are an innate immune cell subset with potent antiviral activity. NK cells are generally categorized as either CD56^bright^ CD16^−^ or CD56^dim^CD16^+^ (96). CD56^bright^CD16^−^ NK cells are often poorly cytolytic but can secrete inflammatory cytokines such as interferon-γ and TNF-α. CD56^dim^ CD16^+^ NK cells express high levels of perforin and granzyme-B and are highly cytolytic (96). NK cells can lyse virally infected cells by two main mechanisms: lysis of cells expressing stress receptors and/or expressing low levels of MHC class I or lysis of cells *via* antibody-dependent cell-mediated cytotoxicity (ADCC). In the former case, a delicate balance of activating (i.e., NKG2D) and inhibitory receptors [i.e., killer-cell immunoglobulin-like receptors (KIRs)] determines the cytolytic potential of NK cells. During HIV infection, Nef mediates MHC-I downregulation to avoid CTL killing (97). This can predispose HIV-infected cells to NK cell-mediated lysis, as MHC-I interacts with inhibitory KIRs. In the case of ADCC, antibody binding to Fc receptors (i.e., CD16) on NK cells results in perforin and granzyme release and cytotoxicity.

In both healthy and chronically HIV-infected humans, NK cells represent a very small percent of cells in the LN (<3%) (98). However, during chronic HIV infection, the CD56^dim^CD16^+^ subset becomes depleted and a novel CD56^−^CD16^+^ subset emerges (99). CD56^−^CD16^+^ NK cells appeared to be poorly cytolytic and possibly anergic (99). Similar observations have been made in SIV infection. During chronic SIV infection, the frequency of NK cells in LNs was approximately equal to naïve animals. However, during chronic infection, the percent of CD56^−^CD16^+^ NK cells was higher. As in HIV infection, CD56^−^CD16^+^ NK cells generated during SIV infection had limited cytotoxic activity and expressed a marker of anergy/exhaustion (100). These data suggest that LN NK cells may become exhausted/anergic during chronic infection.

Recently, it was reported that NK cells enter the B cell follicle and control SIV infection in African green monkeys (AGMs) (101). Unlike rhesus macaques, AGMs usually do not develop SAIDs, exhibit effective control of SIV in SLOs, and do not accumulate SIV virions on FDCs (102–104). During SIV infection, rhesus macaques exhibited a gradual decline in NK cell frequencies in LNs, while AGMs NK frequencies stayed relatively constant (101). Importantly, the numbers of NK cells in B cell follicles increased during SIV infection of AGMs, but not of rhesus macaques (101). SIV DNA levels had a strong negative correlation with frequencies of NK cells in the LN in AGM, but not in rhesus macaques. In AGM, NK cell localization to B cell follicles appeared to be associated with IL-15 production by FDCs. IL-15 is known to be essential for NK cell survival. Furthermore, anti-IL-15 treatment of AGMs resulted in near total depletion of NK cells from blood and LNs. This treatment also resulted in a large increase in plasma viremia and RNA^+^ and DNA^+^ cells in LNs including in the B cell follicles, suggesting that NK cells were critical to controlling SIV replication in the B cell follicle. However, it is important to note that anti-IL-15 treatment likely affects more than just NK cells. Notably, some decrease in blood CD8^+^ T cells was observed after anti-IL-15 treatment (101). So, while not completely definitive, this report provides strong evidence that the effective control of SIV infection in AGMs may be largely mediated by the ability of NK cells to efficiently infiltrate B cell follicles.

## Cure Strategies and the B-Cell Follicle

A wide variety of strategies have been proposed to cure HIV. In this review, we will discuss many of the prevailing cure strategies and their potential to clear the HIV reservoir in the B cell follicle (Table 1). While clearing HIV reservoirs in the B-cell follicle is likely necessary for the development of an effective cure, it may not be sufficient. Other anatomical sites have been proposed to be sites of persistence, most notably the central nervous system (105). However, strategies to target these reservoirs are out of the scope of this review.

**Table 1:** Summary of HIV cure strategies discussed in this review.

Cure strategy	Pros	Cons	Engineering to target B cell follicle	Ability to clear HIV on follicular dendritic cell (FDC) network
CTL expanded *ex vivo*	May not require transduction	Cost of leukapheresis; risk of emergence of escape variants	Cytokine treatment or transduction to express CXCR5	Likely ineffective due to lack of presentation of HIV peptides on MHC-I
Chimeric antigen receptor T cells	Ligands are not MHC-I dependent, low risk of escape variants, demonstrated long-term persistence and safety	Cost of leukapheresis; requires transduction of large numbers of lymphocytes	Cytokine treatment or transduction to express CXCR5	Unknown, but possible due to MHC-I independence
Immunotoxins	Ligands are not MHC-I dependent	Development of anti-toxin immune responses could reduce efficacy of multiple doses	Form immunotoxin-immune complexes to localize to surface of FDCs	Unknown, but possible due to MHC-I independence
Broadly neutralizing antibodies	High neutralization potential	High risk of escape variants and have proven ineffective at producing durable reductions in viremia	Transduce CXCR5^+^ cells to express the antibodies *in vivo*	Unknown, but possible due to MHC-I independence
Bispecific antibodies	High neutralization potential also can function as a latency reversal agent (LRA)	Risks of escape variants and expensive to produce *ex vivo*	Transduce CXCR5^+^ cells to express the antibodies *in vivo*	Unknown, but possible due to MHC-I independence
Rituximab	Demonstrated ability to destroy B cell follicles	May induce immunodeficiency	N/A	Would likely destroy most or all FDCs
Histone deacetylase inhibitors and protein kinase C agonists	May be combined with other strategies to improve efficacy	Have failed to produce durable remission in cure trials when used alone. Some may inhibit CTL responses	N/A	Likely ineffective because FDCs are not infected
Recombinant IL-15/ALT-803	Acts as an LRA and can increase CTL responses. May be combined with other strategies to improve efficacy	Multiple doses in quick succession have diminishing effects	N/A	Unknown, LRA activity ineffective as FDCs are not infected, but proinflammatory effects could have some effect on FDC-bound HIV

*The pros, cons, potential B cell follicle engineering strategies, and ability to clear HIV particles on the FDC network are listed. N/A = not applicable*.

A minority population of HIV-infected individuals, known as elite controllers, are able to suppress HIV replication effectively and maintain undetectable viral loads for years, although virus can usually be identified in them using sensitive techniques (106). It has been demonstrated that the early initiation of ART after primary infection can lead to effective long-term control of viral replication without continuing ART (107, 108). However, this effect is only seen in a subset of patients and is dependent on beginning treatment very soon after initial infection, and is usually not durable over more than a few years (109).

Furthermore, these patients still have a readily detectable HIV reservoir and are therefore considered to have a functional and not sterilizing cure (108). The only known person to have a possible sterilizing cure with no detectable reservoir is Timothy Brown (a.k.a. the Berlin patient). After being diagnosed with acute myeloid leukemia, the Berlin patient received chemotherapy and whole body irradiation followed by a stem cell transplant from a donor who was homozygous for the CCR5 delta32 allele (110, 111). Initial reports failed to detect any HIV DNA or RNA in the Berlin patient (110, 111). However, one report has suggested that he may still harbor extremely low levels of HIV RNA and DNA. It is important to note that the levels of HIV RNA and DNA were so low, it is possible they were false positives (112). Regardless of the true nature of the Berlin patient’s cure, his case stands as an important milestone in HIV cure research. Importantly, bone marrow transplant patients who did not receive CCR5 homozygotic donor cells have all relapsed, although in some instances the timing of the relapse was delayed relative to what is seen in HIV-infected individuals who stop ART (113). Residual latently infected CD4^+^ T cells are frequently invoked as the source of these relapses; however, the role of FDC-bound virions cannot be excluded, particularly since they are radiation resistant (114, 115).

Given that CTL are critical for controlling viremia, several proposed cure strategies revolve around altering or expanding CTL. One proposal is to isolate CTL from infected patients and expand HIV-specific CTL *ex vivo* using HIV peptides and then reinfuse these back into patients. Several versions of this strategy have been tried over the years, but have failed to yield large or durable reductions in viremia (116–118). One problem associated with expanded CTL is that escape mutants can arise (117). Attempts to circumvent this problem by expanding polyclonal CTL against multiple HIV epitopes have been attempted, but to date have been unable to achieve durable reductions in viremia (116, 119). A similar strategy is to use chimeric antigen receptor (CAR) T cells that target HIV moieties. Several CARs have been developed that use the CD4 ectodomain and/or anti-ENV single chain antibody fragments to bind HIV envelope (120–122). CAR T cells have several potential advantages over *ex vivo* expanded CTL. CAR T cells do not need to recognize their ligands in the context of MHC, meaning that Nef-mediated downregulation of MHC-I should not affect CAR T cell killing. Additionally, CAR T cells can target highly conserved regions of HIV envelope such as the CD4 binding site, making escape mutations less likely. In a clinical trial, a CD4 ectodomain-based CAR resulted in only minor reductions in HIV reservoir size (123). However, an encouraging observation with these studies was that the CAR T cells were able to persist in many patients for over 10 years, demonstrating that durable engraftment of CAR T cells is achievable (124). Additionally, many improvements have been made in HIV-specific CAR T cells since those studies were undertaken, leaving CAR T cell therapies as a very promising avenue to be explored.

Critically, in the case of either *ex vivo* expanded CTL or CAR T cells, penetration into the B cell follicle will likely be poor without efforts to target these cells to the B cell follicle. In order to target CTL or CAR T cells to the B cell follicle, it may be necessary to treat the cells with cytokines or transduce them to constitutively express the follicular homing molecule CXCR5. One recent study has demonstrated the ability of TGF-β to induce CXCR5 expression in CTL (125). Transduction with CXCR5 has been demonstrated to allow the B-follicular localization of CTL in rhesus macaques, but it remains undetermined if they retain effective antiviral responses and will be able to clear the B-follicular HIV reservoir (126).

A similar strategy is to use HIV-specific immunotoxins, formed by either an anti-ENV single-chain antibody fragment or the CD4 ectodomain conjugated to the translocation and effector domain of a bacterial toxin, such as *Pseudomonas aeruginosa* exotoxin A (127). An anti-HIV immunotoxin in combination with ART was shown to be very effective at reducing HIV RNA^+^ cells in bone marrow–liver–thymus (BLT) humanized mice (128). However, BLT humanized mice do not effectively form B cell follicles or GCs during immune reconstitution (129). To date, no strategies to target anti-HIV immunotoxins to the B cell follicle have been reported. Therefore, it remains an open question if immunotoxins can effectively deplete HIV-infected cells in the B cell follicle. However, it is known that HIV-ICs will accumulate on FDCs in B cell follicles and remain potently infectious long term (75). Perhaps treatment of immuntoxins with non-neutralizing antibodies, would result in the decoration of FDCs with viable immunotoxin-ICs that would retain the ability to clear HIV-infected cells, but this remains untested.

Broadly neutralizing antibodies have been proposed and tested for HIV cure, with the idea that immune cells, mainly NK cells, would kill infected cells through ADCC against HIV envelope protein. Previous attempts have shown reductions in viremia, but failed to garner durable responses (130). Now attempts are being made to engineer bispecific antibodies to increase the number of strains that can be neutralized (131). Recently, a bispecific antibody that targets CD3 and gp120 (CD3/VRC07) has been developed (132). The CD3/VRC07 antibody can both act as a latency reversal agent (LRA) by stimulating latently infected T-cells through CD3 binding and facilitate ADCC against cells expressing newly synthesized gp120. Furthermore, the CD3/VRC07 antibody was recently demonstrated to be able to induce follicular CD8^+^ T cell-mediated killing of infected cells *ex vivo* (67). Again, it remains unclear how effective any antibody-based strategy would be against the HIV reservoir in the B cell follicle. However, CXCR5 expressing lymphocytes could be transduced to secrete the bispecific antibodies to help localize the effects to the follicle. Given the recent demonstration of highly effective control of SIV infection in B cell follicles by CXCR5^+^ NK cells in AGMs and the high ADCC potential of NK cells, combining strategies to localize NK cells to B cell follicles with anti-HIV antibodies may prove highly effective.

Another potential strategy to deplete the HIV reservoir in the B-cell follicle is to destroy B cell follicles with B cell targeting agents such as rituximab. Rituximab is an anti-CD20 antibody fragment that induces apoptosis in B cells. Depletion of B cells with rituximab in rhesus macaques prior to and during infection with SIV resulted in no statistically significant differences in viral load during either acute or during chronic infection (between 0 and 240 dpi), although viral loads trended lower in rituximab treated macaques (70). Interestingly, in follow-up studies (>400 dpi) SIV RNA was undetectable in 6 of 7 rituximab treated animals, while SIV RNA was detectable in four of four control animals (70). These data may suggest that rituximab treatment can contribute to control of viremia. However, it is important to note that B cell depletion was incomplete in three of seven animals, and these animals had no delay in seroconversion (70). Additionally, the animals were infected with an SIV strain that is highly prone to neutralization. Importantly, animals with complete B cell depletion showed no evidence of SIV virions accumulating on FDCs in LNs or Peyer’s patches at 28 dpi (70). This demonstrates the potential of rituximab to prevent the accumulation of SIV-ICs on FDCs. However, anti-FDC stains were not reported and as the B cell depletion greatly inhibited the production of antibodies, IC formation was also likely inhibited. Therefore, it is unclear if existing FDCs were eliminated or if they couldn’t bind SIV virions due to lack of IC formation. It also remains unknown what effect rituximab treatment will have on SIV/HIV-ICs already bound to FDCs. Finally, none of the animals were ART treated. Any cure strategy utilizing rituximab would likely occur during ART. It is also important to consider that a possible complication of rituximab treatment would be immunodeficiency and/or immunopathologies.

### Latency Reversal Agents

All the cure strategies discussed so far rely upon active transcription and translation of viral products. Therefore, any successful cure strategy will almost certainly need to be combined with an effective LRA. An in-depth discussion of the wide variety of potential LRAs is outside the scope of this review. However, three promising candidates worth mention are protein kinase C (PKC) agonists, histone deacetylase inhibitors (HDACi), and recombinant IL-15 (or an IL-15 superagonist such as ALT-803).

One of the most promising classes of LRAs is PKC agonists. PKC agonists are believed to cause latency reversal by promoting NF-κB nuclear translocation and binding to HIV LTR (133, 134). PKC agonists can effectively activate HIV transcription across a wide variety of HIV latency models (135). Importantly, in a model of *ex vivo* latency reversal from resting CD4 T cells from infected patients, only the PKC agonist Bryostatin 1 was able to induce HIV mRNA transcription (136). Recently, it was demonstrated that a synthetic analog of Bryostatin 1 could induce HIV activation in the humanized BLT mouse model (137). However, as previously mentioned, the BLT mouse model does not effectively recapitulate B cell follicles and GCs (129). In summary, PKC agonists are a promising class of LRAs to be used in cure strategies. However, to our knowledge, the ability of PKC agonists to activate HIV reservoirs in the B cell follicle remains untested.

One of the most extensively investigated strategies to date to activate and eliminate the latent HIV reservoir has been the use of HDACi. Sung et al. demonstrated that HIV-specific CD8^+^ T cells effectively kill cells in which latent virus has been reactivated by HDACi *in vitro* (138). Nevertheless, although increases in vRNA have been documented in ART-treated humans who received HDACi, no decreases in latently infected cells have been observed *in vivo* (139–143). Similarly, in ART-treated SIV-infected rhesus macaques, treatment with the HDACi romidepsin resulted in reproducible induction of plasma viremia, but no delays in rebound viremia upon cessation of ART (144). Although some have suggested that the failure of HDACi to diminish the latent reservoir or delay rebound viremia is due to the lack of effector virus-specific CD8^+^ T cells (145), CD8 T cell depletion in ART-treated rhesus macaques has demonstrated that they contribute to virologic control even during ART (146). Alternatively, the failure of HDACi to diminish the latent reservoir may be due to the failure of virus-specific CD8^+^ T cells to access B cell follicles in large numbers, where the majority of vRNA^+^ cells are located in treated disease (60). Therefore, while HDACi have been demonstrated to be ineffective in diminishing the latent reservoir alone, HDACi may be more effective when combined with other cure strategies described in this review.

Both recombinant IL-15 and the IL-15 superagonist ALT-803 have been demonstrated to have latency-reversing properties (147). Importantly, ALT-803 also enhances CTL function against productively infected cells (147). Some other potential LRAs, including a subset of HDACi, inhibit CTL effector functions (148). Although, it is worth noting that the *in vivo* significance of HDACi-mediated CTL inhibition is a subject of continuing debate (149). It was recently reported that in SIV-infected rhesus macaques, administration of ALT-803 resulted in potent (~2 log) reductions in viremia (150). However, these reductions were transient and repeated doses in close succession (2 weeks apart) had diminishing effects on viral replication. A repeat dose given after a long break (29 weeks later) demonstrated renewed reductions in viral replication (150). These data strongly suggest that ALT-803 has potent antiviral effects *in vivo*, but is ineffective alone in controlling replication and its antiviral effect has limited durability. The lack of CTL inhibition, combined with the importance of IL-15 in mediating follicular SIV control by NK cells in the AGM model makes ALT-803 a particularly promising candidate as an LRA (101). However, it remains to be seen what effects ALT-803 will have on the follicular reservoir and if targeting ALT-803 to the B cell follicle will be necessary.

### Clearing the HIV Reservoir on FDCs

It is unclear how any of the cure strategies described above will affect the FDC-bound HIV reservoir. CTLs expressing TCRs against HIV peptides would presumably be totally ineffective against FDC-bound HIV as the FDCs themselves do not become infected and thus likely do not express HIV peptides on MHC-I. A more promising avenue is CAR T cell therapies because they are independent of MHC-I presentation. However, the stability of FDC-bound HIV virions may suggest that they are largely shielded from sCD4-mediated gp120 shedding. If true this could mean that CD4-based CAR T cells may be unable to bind FDC-bound HIV virions. However, anti-CD4 antibodies prevent infection of CD4 T cells by FDC-bound HIV-ICs *ex vivo*, suggesting that the CD4 binding site on gp120 must become at least temporarily exposed on FDCs (76). Furthermore, even if CAR T cells can initiate an immune response against FDC-bound HIV virions, it is unclear how effective this would be in removing the reservoir. Given the dendritic morphology of FDCs, it remains unknown if a CTL attack on the extremities of these dendrites will kill the FDC or just damage a single dendrite. For all of these reasons, it remains an open question whether CD4 CAR T cell therapies will remove or significantly reduce the FDC-bound HIV reservoir. These problems may also apply to strategies that rely on ADCC.

It is equally unclear if immunotoxins will be able to kill FDCs decorated with HIV-ICs. If it is true that HIV-ICs are endocytosed by the FDC, then it is possible that immunotoxins could effectively kill FDCs by delivering the toxic moieties directly to the cytoplasm of the cell. However, these endocytic compartments, if they exist *in vivo*, are believed to be non-degradative and thus presumably non-acidic. Many bacterial toxins require endosomal acidification for membrane insertion and intoxication (151, 152). Furthermore, many bacterial toxins, such as PE, require retrograde trafficking to the endoplasmic recticulum (ER) and it is unclear if HIV-IC recycling endosomes could be diverted to the ER or any other subcellular compartments (152). For these reasons, it remains unclear if immunotoxins can efficiently kill FDCs by binding to HIV-ICs on the cell surface.

It may be possible to specifically dislodge HIV-ICs from FDCs by targeting the Fc and complement receptors on the FDC surface that bind HIV-ICs. Treatment with the ectodomain of the complement receptor fused to an Fc domain (CD21-Fc) was able to significantly reduce the number of virions bound to FDC *in vitro*, but as yet remains untested in any *in vivo* model (80). It is possible that specific clearing of HIV-ICs from the surface of FDCs or targeted killing of FDCs coated with HIV-ICs will prove unachievable *in vivo*. If this is the case, total depletion of the B cell follicle and FDCs by rituximab or some other agent may be necessary to remove the FDC-bound HIV reservoir.

## Concluding Remarks

**Figure 1:**
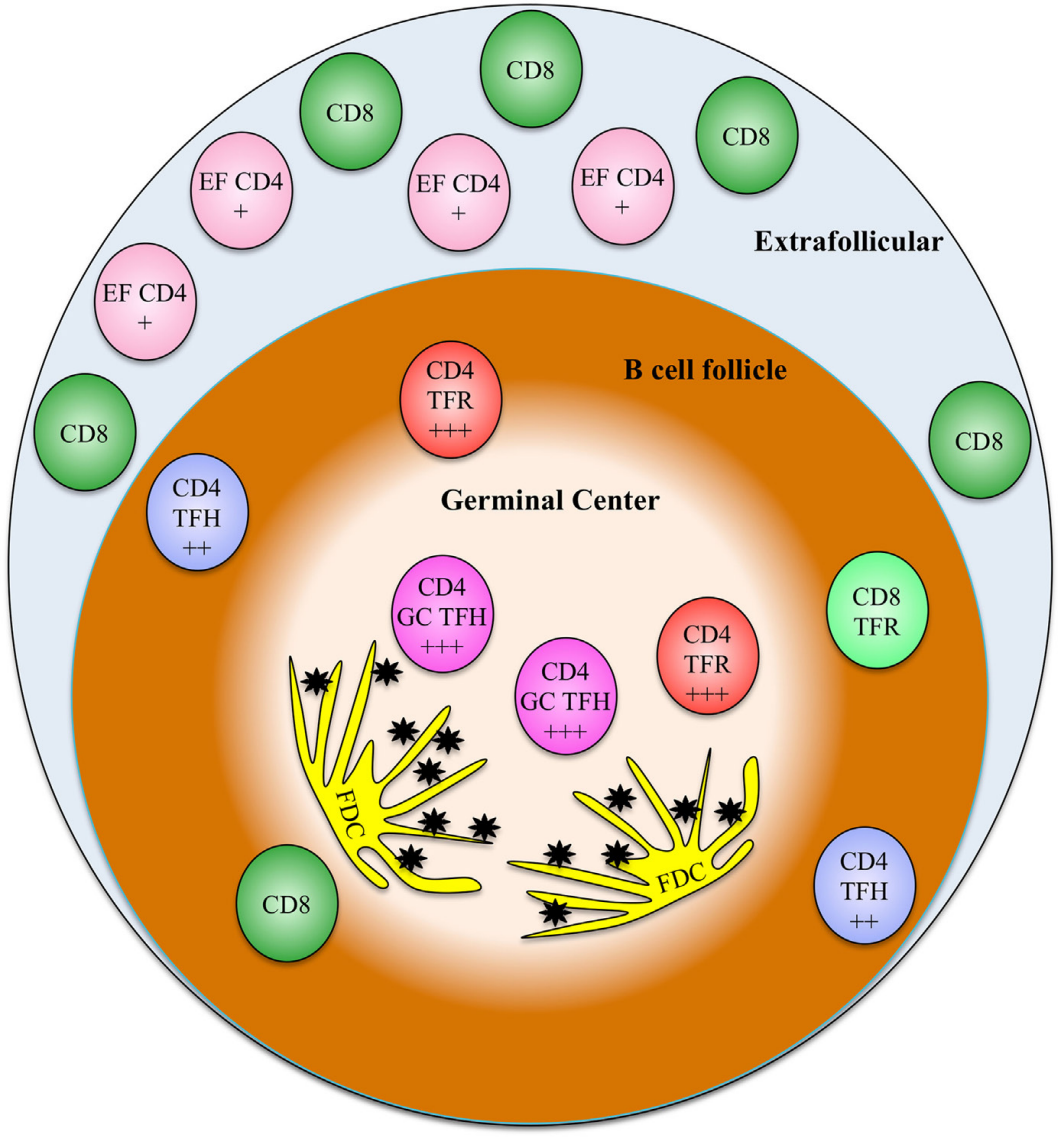
A model showing the relative frequencies and localizations of various relevant T cell types discussed in this review. The relative susceptibility of CD4 subsets to HIV infection is indicated on each cell type (+ indicates somewhat susceptible, + + + indicates highly susceptible). EF CD4, extrafollicular CD4 T cells; TFH, T follicular helper cells; GC TFH, germinal center T follicular helper cells; TFR, T follicular regulatory cells; FDC, follicular dendritic cells, black stars represent extracellular HIV immune complexes.

The susceptibility of follicular CD4^+^ T cell subsets, poor follicular CTL accumulation and possibly function, a large extracellular FDC-bound viral reservoir, and possibly other factors all promote the B cell follicle as a critical sanctuary for HIV replication and persistence (Figure 1). HIV replication in the B cell follicle also likely mediates defects in humoral immunity that promote systemic defects in anti-HIV immunity. Targeting follicular reservoir of virus will likely be essential to suppression or eradication of HIV. Thus, a better understanding of the mechanisms mediating HIV persistence in the B cell follicle is critical to development of an effective HIV cure strategy.

## Author Contributions

MB and EC composed and edited this review. PS contributed to the editing of this review.

## Conflict of Interest Statement

The authors declare that the research was conducted in the absence of any commercial or financial relationships that could be construed as a potential conflict of interest.
